# Supplementary material for: One-Step Oxidation of Orange Peel Waste to Carbon Feedstock for Bacterial Production of Polyhydroxybutyrate
Source: Polymers (Basel). 2023 Jan 30;15(3):697. doi: 10.3390/polym15030697 (PMC9920450; doi:10.3390/polym15030697)
Supplement: Supplementary file 1 [file polymers-15-00697-s001.zip › polymers-2107085-supplementary.pdf]

One-Step Oxidation of Orange Peel Waste to Carbon Feedstock for Bacterial Production of Polyhydroxy-butyrate

Maryam Davaritouchaee[1], Imann Mosleh[1], Younas Dadmohammadi[1], Alireza Abbaspourrad[1]*

[1] Department of Food Science, College of Agriculture & Life Sciences, Cornell University, Stocking Hall, Ithaca, New York, 14853, United States

*Corresponding Author, Email address: Alireza@cornell.edu

Table S1: ANOVA study of effect of each variables (carbon source and time) on response (CDW and PHB content), and the interactions of variables

Response Cell Dry Weigh

**Source**	**Nparm**	**DF**	**Sum of Squares**	**F Ratio**	**Prob > F**
Carbon Source	5	5	23.263927	84.3833	<.0001*
Time (h)	3	3	5.116962	30.9339	<.0001*
Carbon Source*Time (h)	15	15	3.279592	3.9653	0.0002*

Response PHB Content

**Source**	**Nparm**	**DF**	**Sum of Squares**	**F Ratio**	**Prob > F**
Carbon Source	5	5	1081918.1	193.4373	<.0001*
Time (h)	3	3	384803.7	114.6658	<.0001*
Carbon Source*Time (h)	15	15	485103.7	28.9107	<.0001*

Table S2: the yields of fermentable sugar monomer and inhibitory compounds after 1, 2, and 3 h of oxidation in each treatment run

		Control	0.02-OX-OP	0.05-OX-OP	0.25-OX-OP	0.5-OX-OP	1-OX-OP
1 h	Glucose	31.31	48.62	55.94	36.97	15.94	17.55
	Galactose	NA	1.90	3.12	1.69	0.99	1.68
	Xylose	NA	2.48	2.70	1.36	0.93	1.51
	Fructose	8.58	22.34	24.84	13.66	11.27	11.26
	Acetic Acid	0.08	0.56	0.52	0.44	1.40	1.01
	HMF	NA	-1.74	-1.54	-1.25	-1.05	-1.62
	Furfural	NA	0.12	NA	NA	NA	0.15
	Galacturonic acid	45.83	26.48	22.29	19.20	18.25	9.11
2 h	Glucose	30.63	47.55	32.42	24.58	10.01	9.34
	Galactose	NA	NA	NA	NA	1.11	2.21
	Xylose	NA	NA	NA	NA	0.91	1.64
	Fructose	6.19	16.37	10.86	11.79	5.80	10.07
	Acetic Acid	#DIV/0!	0.44	0.62	0.84	0.51	0.57
	HMF	#DIV/0!	-1.70	-1.50	-1.33	-1.36	-1.59
	Ferfural	#DIV/0!	0.17	0.15	0.12	0.11	0.10
	Galacturonic acid	47.54	22.74	13.08	13.12	13.03	6.27
3 h	Glucose	31.52	44.01	30.66	20.97	5.71	9.34
	Galactose	NA	NA	NA	NA	0.98	2.77
	Xylose	NA	NA	NA	NA	0.98	2.77
	Fructose	6.42	14.95	9.71	11.78	5.60	8.41
	Acetic Acid	NA	NA	0.38	0.61	1.62	1.29
	HMF	NA	NA	-1.77	-1.43	-1.49	-1.94
	Ferfural	NA	0.19	0.08	0.08	NA	0.12
	Galacturonic acid	46.34	NA	NA	NA	NA	-1.87

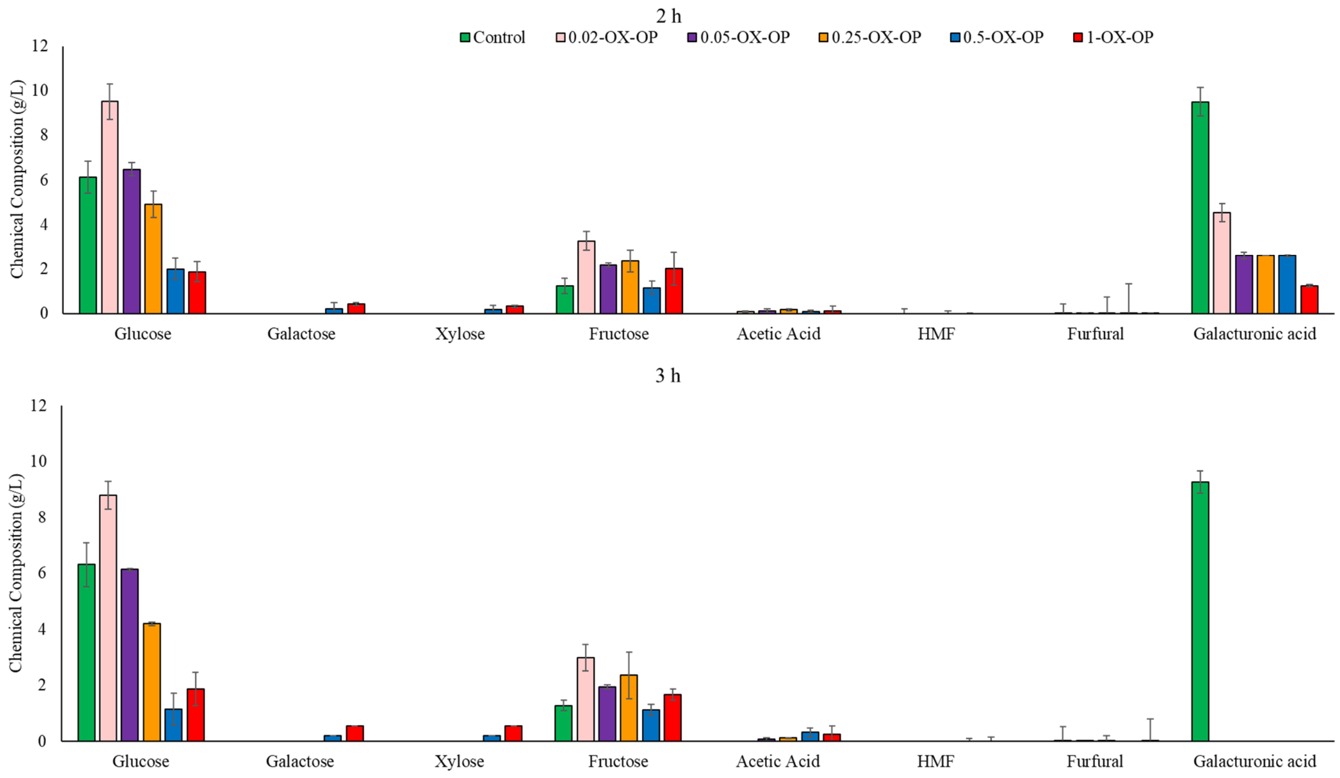

Figure S1: Sugar content in control and pretreated samples for 2 and 3 h of oxidation reaction by different concentration of oxidative agents; control (alkaline aqueous solution without exogenous addition of KO_2), 0.05-OX-OP (orange peels oxidized by 0.05 M KO_2), 0.25-OX-OP (orange peels oxidized by 0.25 M KO_2), 0.5-OX-OP (orange peels oxidized by 0.5 M KO_2), 1-OX-OP (orange peels oxidized by 1 M KO_2)

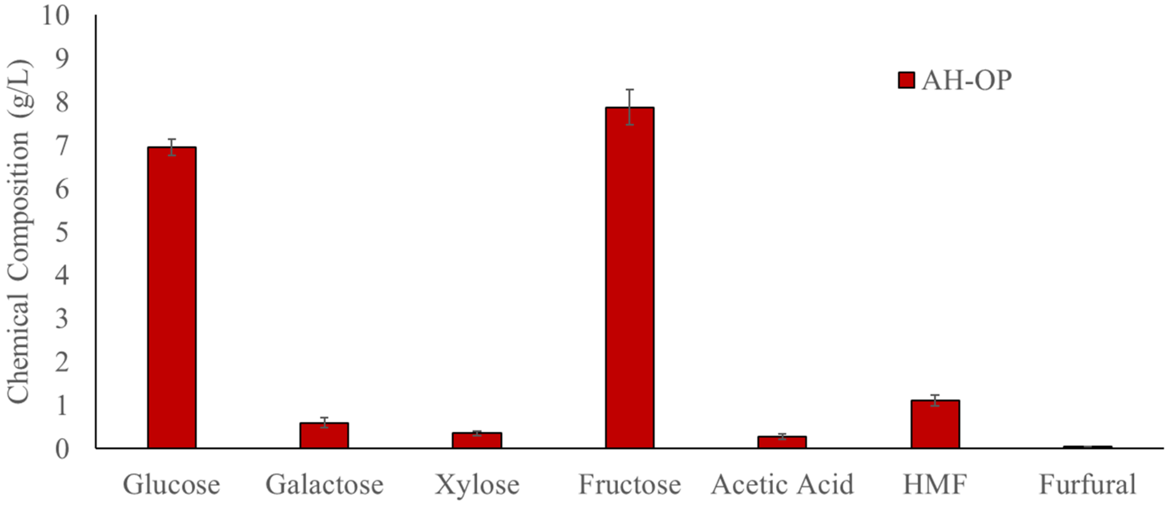

Figure S2: Sugars content of acid hydrolyzed orange peel, 15.77 gr/L released sugar content.

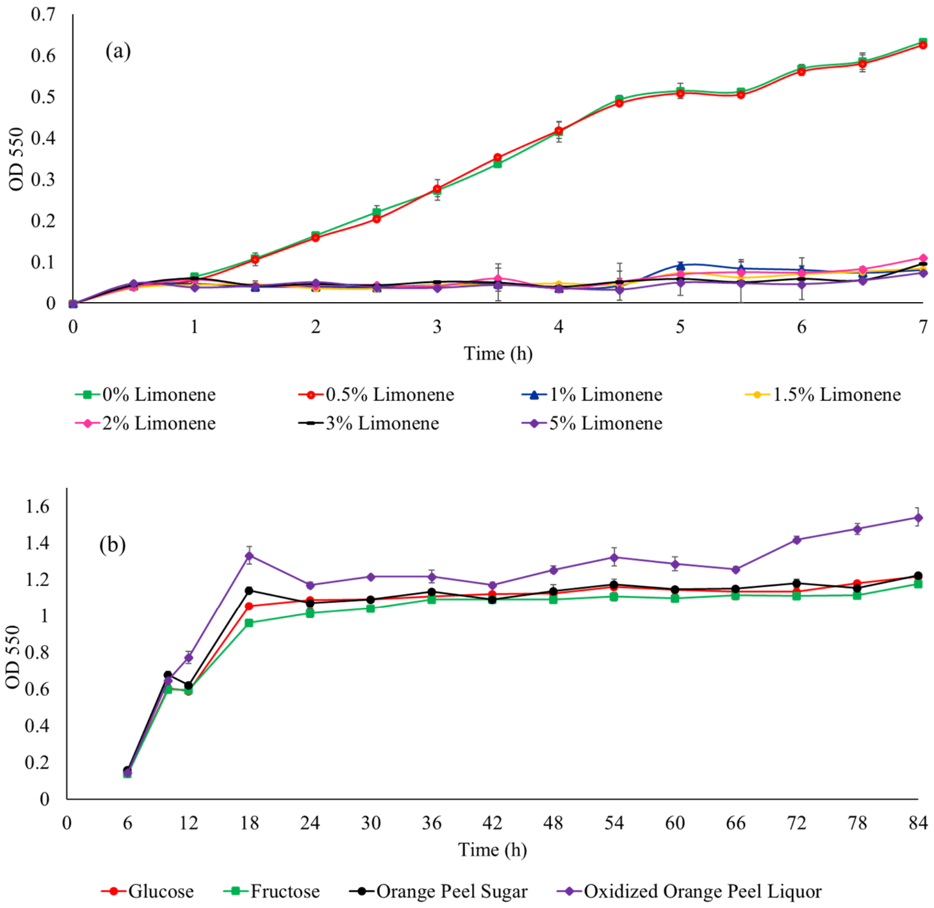

Figure S3: Growth rate of JM109 E. coli in the presence of (a) 0-5% v/v limonene. (b) Bacteria growth comparison when fed with glucose, fructose, simulated orange peel sugar, and oxidized orange peel sugar during 3 days of fermentation.

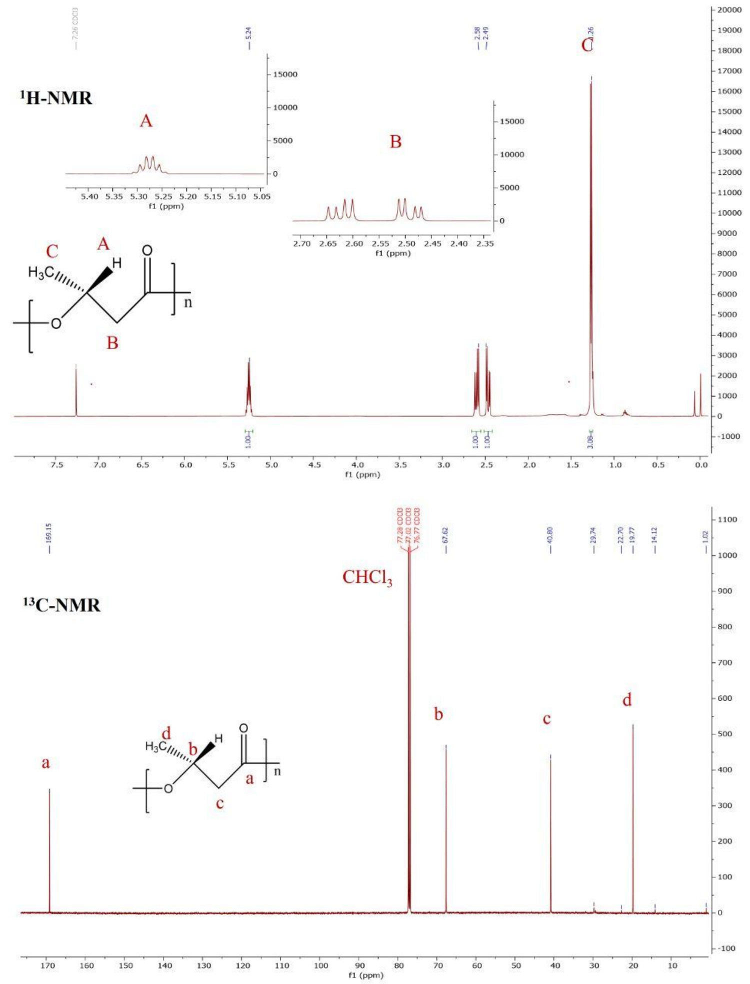

Figure S4: ^{1}H-NMR (up) and ^{13}C-NMR (down) spectra in $CDCl_3$ of PHB derived from cells fed with oxidized orange peels after 72 h.

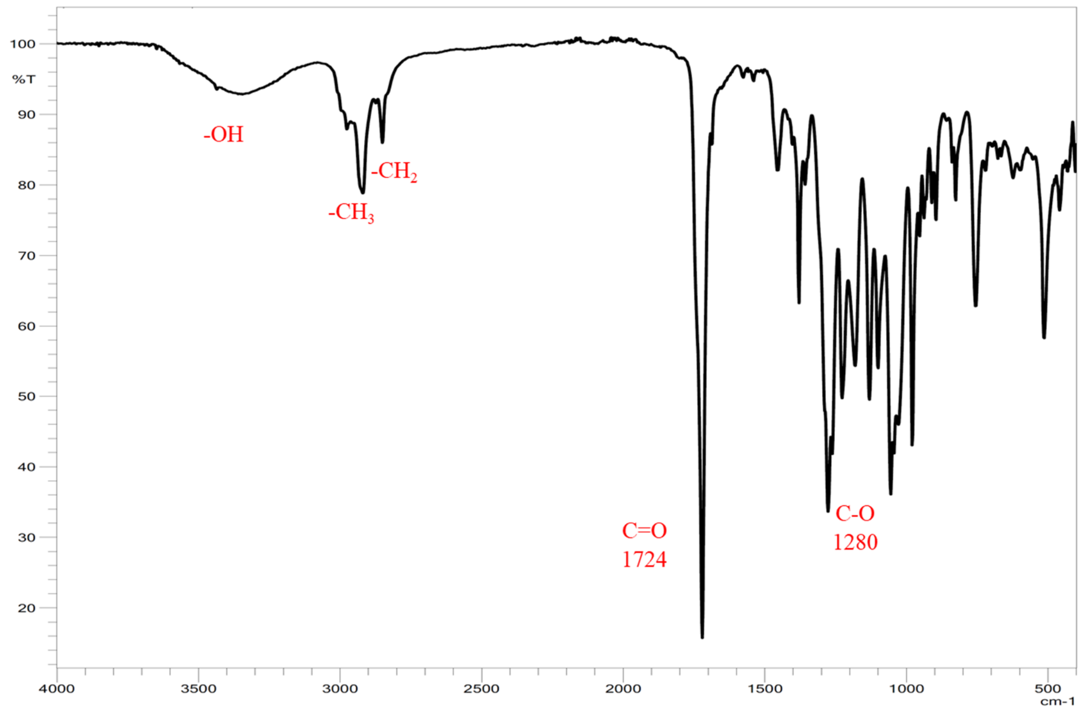

Figure S5: FT-IR spectrum of PHB derived from cells fed with oxidized orange peels after 72 h incubation.

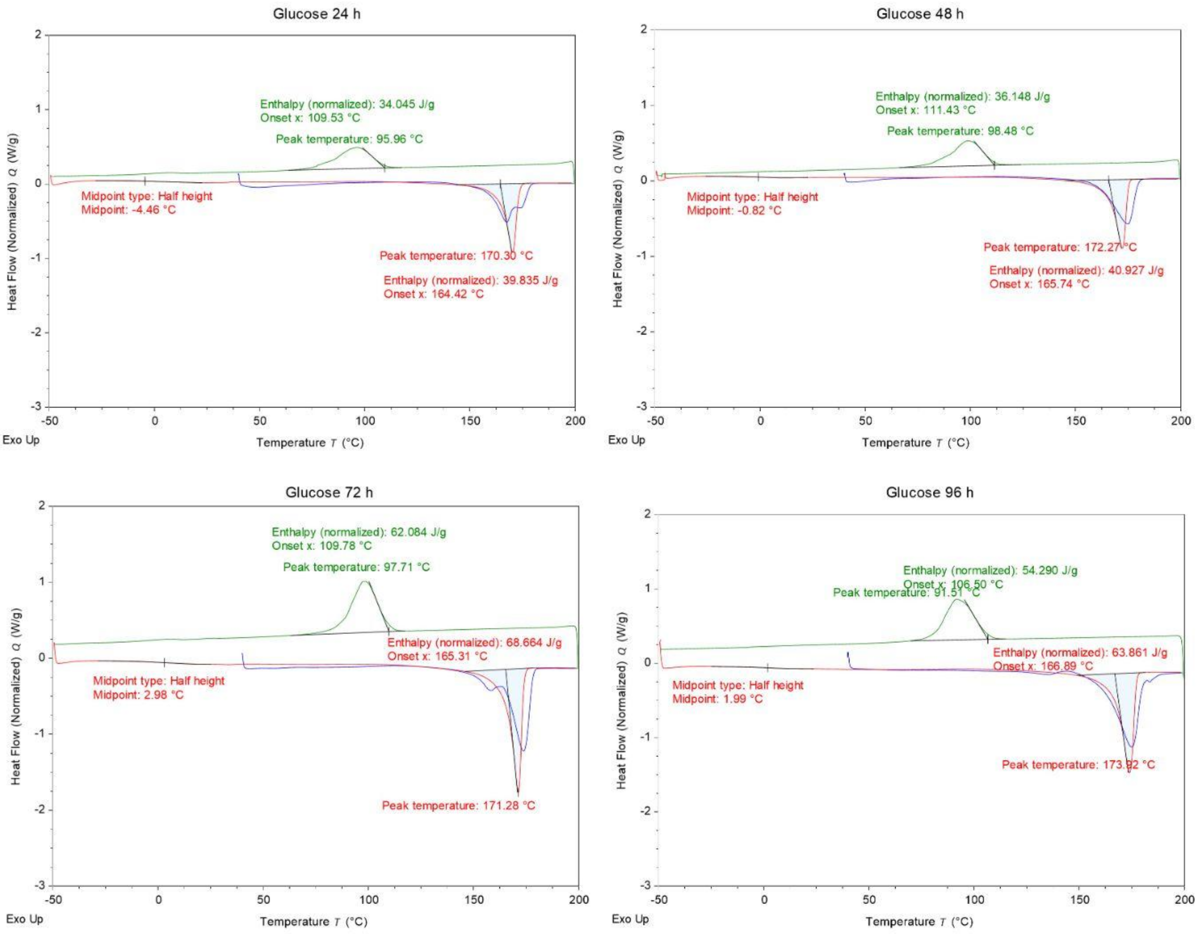

Figure S6: DSC thermograms of produced PHB using glucose as carbon source at designated time frames. Blue curve: the first heating step, green curve: is the cooling step, and red curve: the second heating step.

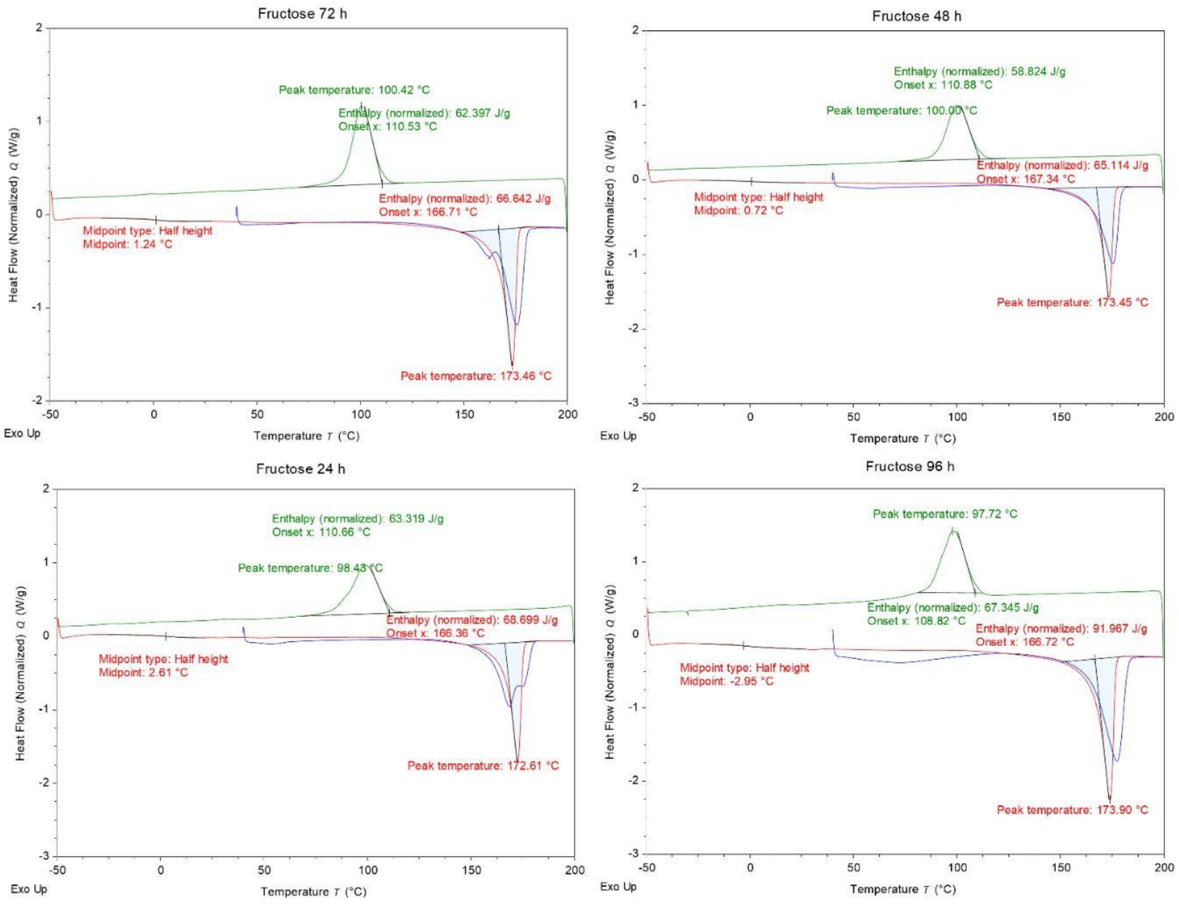

Figure S7: DSC thermograms of produced PHB using fructose as carbon source at designated time frames. Blue curve: the first heating step, green curve: is the cooling step, and red curve: the second heating step.

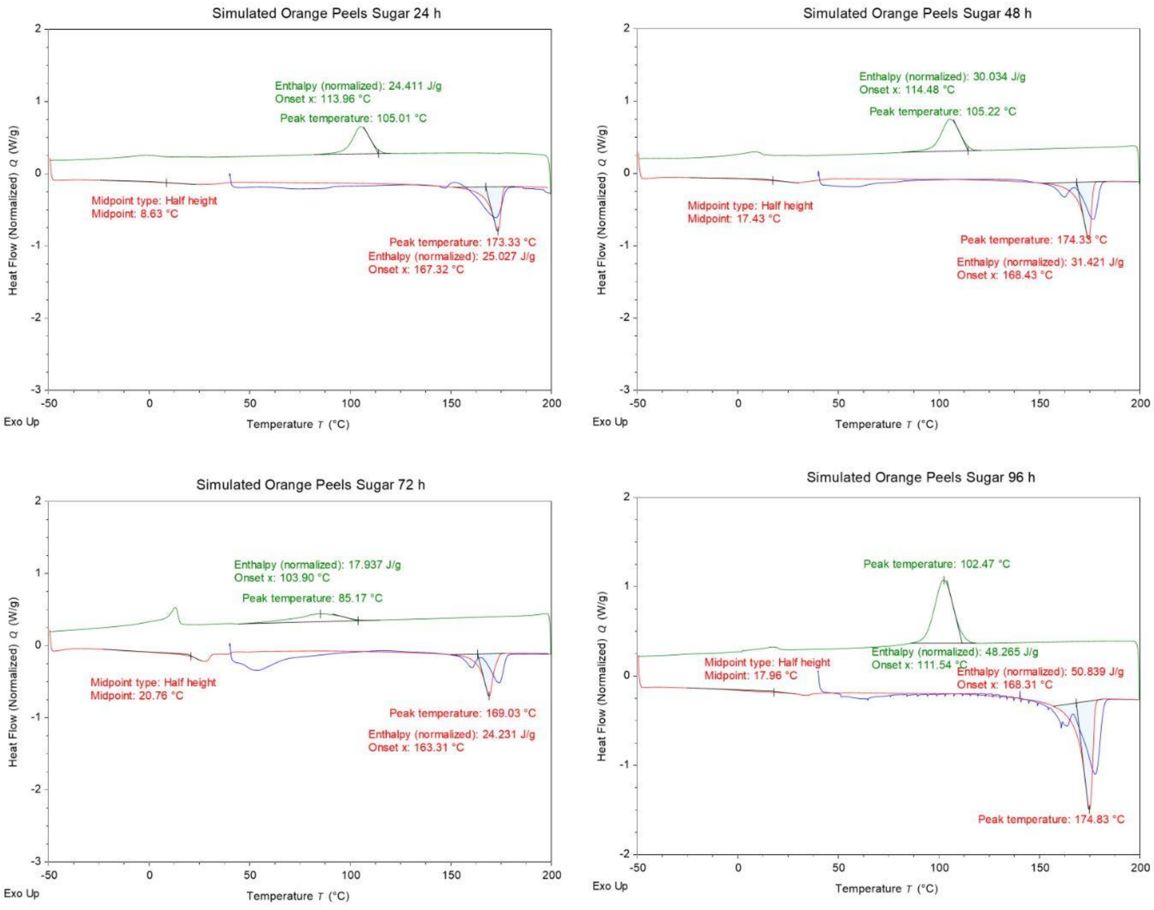

Figure S8: DSC thermograms of produced PHB using simulated orange sugar peels as carbon source at designated time frames. Blue curve: the first heating step, green curve: is the cooling step, and red curve: the second heating step.

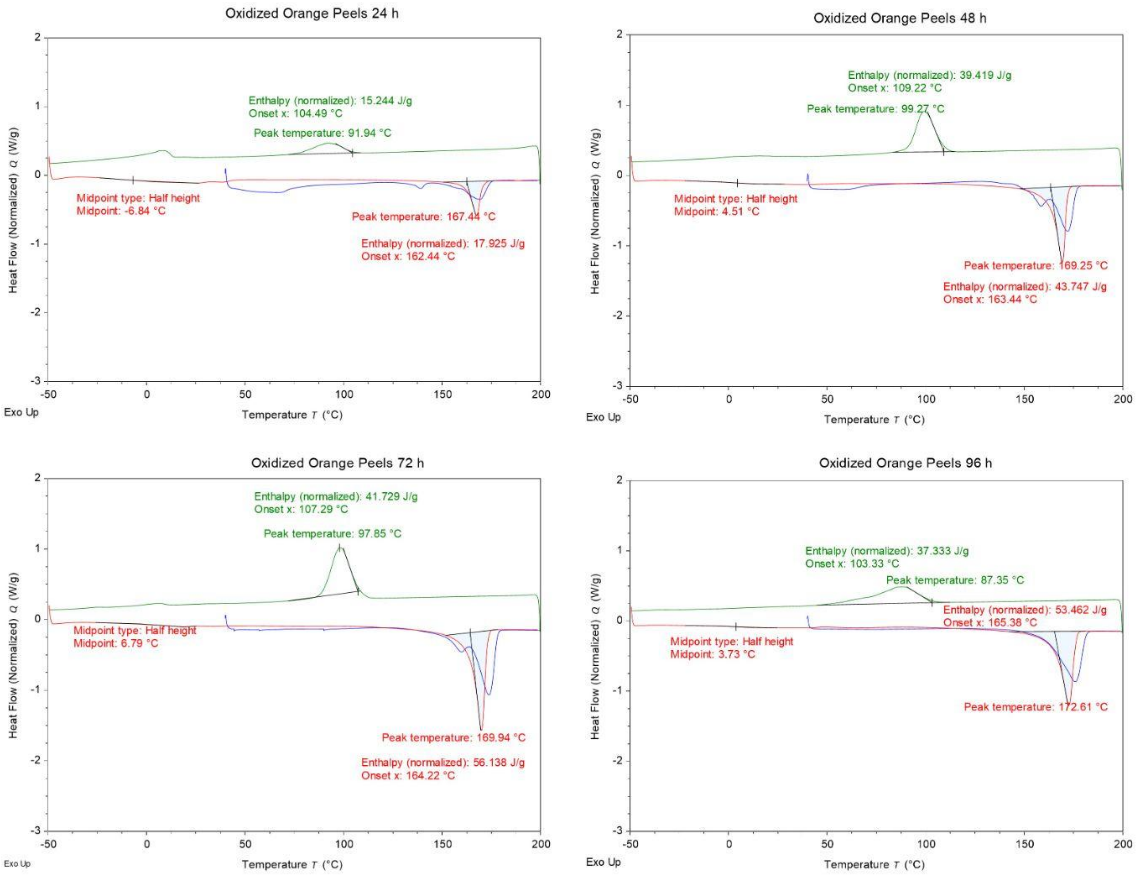

Figure S9: DSC thermograms of produced PHB using oxidized orange peels as carbon source at designated time frames. Blue curve: the first heating step, green curve: is the cooling step, and red curve: the second heating step.
